# Prospects of Therapeutic Target and Directions for Ischemic Stroke

**DOI:** 10.3390/ph14040321

**Published:** 2021-04-01

**Authors:** Jung Hak Kim, So Young Kim, Bokyung Kim, Sang Rae Lee, Sang Hoon Cha, Dong Seok Lee, Hong Jun Lee

**Affiliations:** 1 Research Institute, e-biogen Inc., Seoul 07282, Korea; jnhkim1116@gmail.com; 2 College of Medicine, Chung-Ang University, Seoul 06974, Korea; biochemcau@naver.com; 3 BK21 FOUR KNU Creative BioResearch Group, School of Life Sciences, Kyungpook National University, Daegu 41566, Korea; mideun@knu.ac.kr (B.K.); lee1@knu.ac.kr (D.S.L.); 4 Laboratory Animal Research Center, Department of Pharmacology, School of Medicine, Ajou University, Suwon 16499, Korea; lsr21@ajou.ac.kr; 5 College of Medicine and Medical Research Institute, Chungbuk National University, Cheongju 28644, Korea; shcha@chungbuk.ac.kr; 6 Department of Radiology, Chungbuk National University Hospital, Cheongju 28644, Korea

**Keywords:** ischemic stroke, biomarkers, microglia activation, stem cell therapy, multimodal imaging

## Abstract

Stroke is a serious, adverse neurological event and the third leading cause of death and disability worldwide. Most strokes are caused by a block in cerebral blood flow, resulting in neurological deficits through the death of brain tissue. Recombinant tissue plasminogen activator (rt-PA) is currently the only immediate treatment medication for stroke. The goal of rt-PA administration is to reduce the thrombus and/or embolism via thrombolysis; however, the administration of rt-PA must occur within a very short therapeutic timeframe (3 h to 6 h) after symptom onset. Components of the pathological mechanisms involved in ischemic stroke can be used as potential biomarkers in current treatment. However, none are currently under investigation in clinical trials; thus, further studies investigating biomarkers are needed. After ischemic stroke, microglial cells can be activated and release inflammatory cytokines. These cytokines lead to severe neurotoxicity via the overactivation of microglia in prolonged and lasting insults such as stroke. Thus, the balanced regulation of microglial activation may be necessary for therapy. Stem cell therapy is a promising clinical treatment strategy for ischemic stroke. Stem cells can increase the functional recovery of damaged tissue after post-ischemic stroke through various mechanisms including the secretion of neurotrophic factors, immunomodulation, the stimulation of endogenous neurogenesis, and neovascularization. To investigate the use of stem cell therapy for neurological diseases in preclinical studies, however, it is important to develop imaging technologies that are able to evaluate disease progression and to “chase” (i.e., track or monitor) transplanted stem cells in recipients. Imaging technology development is rapidly advancing, and more sensitive techniques, such as the invasive and non-invasive multimodal techniques, are under development. Here, we summarize the potential risk factors and biomarker treatment strategies, stem cell-based therapy and emerging multimodal imaging techniques in the context of stroke. This current review provides a conceptual framework for considering the therapeutic targets and directions for the treatment of brain dysfunctions, with a particular focus on ischemic stroke.

## 1. Introduction

Stroke is a serious, adverse neurological event and the third leading cause of death and disability worldwide. Recently, the number of stroke patients is increasing due changing lifestyles and the aging population [1]. Most strokes are caused by a block in cerebral blood flow, resulting in neurological deficits through the death of brain tissue. Stroke can be divided into two subtypes: ischemic and hemorrhagic [2,3]. Hemorrhagic stroke is caused by the vascular rupture of a cerebral artery or vein in the brain, causing localized bleeding in the surrounding tissue. Two main types of hemorrhagic stroke have been classified: intracerebral and subarachnoid. Subarachnoid hemorrhage is characterized as a stroke that occurs as a result of saccular aneurysms interrupting the subarachnoid space. On the other hand, hemorrhagic stroke accounts for approximately 10% to 15% of all stroke cases, with a mortality rate of 40% to 50% after the onset of the disease [4]. Ischemic stroke occurs as a result of an obstruction of blood flow in the cerebral artery stemming from a thrombus or embolus, hypoperfusion from decreased blood pressure, or a reduction in oxygen levels due to systemic hypoxia. Collectively these represent the most common causes of cerebrovascular events, such as transient ischemic attack (TIA). Ischemic stroke accounts for 85% to 90% of all types of stroke [5]. Common risk factors for stroke are widely related to hypertension, dyslipidemia, abdominal obesity, alcoholism, heart disorders, diabetes, and smoking, all of which affect a large number of individuals. In addition, other risk factors, including atrial fibrillation, TIA, and the microglia associated inflammatory response are well known risk factors for stroke [6,7,8].

The immediate treatment goals in most ischemic stroke cases are focused on stabilizing the patient and rapidly recovering cerebral blood flow. This requires a quick assessment before treatment and early intervention to restore maximal reperfusion of brain tissue [9].

Previous studies have reported that recombinant tissue plasminogen activator (rt-PA) is the only effective acute treatment option for ischemic stroke patients that has been approved by the United States Food and Drug Administration. The primary mechanism of this agent is the reduction in thrombi and emboli by thrombolysis. However, rt-PA must be administered within a very short therapeutic timeframe (3 h to 6 h) after the onset of acute stroke symptoms [10,11,12,13]. Based on several clinical trials, administration of intravenous rt-PA within 3 h of the onset of symptoms is the recommended method of treatment for eligible patients who experience ischemic stroke.

The goal of endovascular thrombectomy is to expand the thrombolytic therapeutic time after symptom onset by rapidly recanalizing the interrupted blood vessels and restoring tissue perfusion, with the hope that early brain tissue reperfusion can prevent brain cells from being damaged [14,15,16,17,18]. Nevertheless, the widespread use of this medication for thrombolytic therapy is limited and cannot protect against neuronal cell death within the brain tissue during the acute phase of the disease [19]. Moreover, the risk of severe hemorrhagic complications limits rt-PA treatment to a small percentage of ischemic stroke patients [20]. Although neuroprotective therapies, and other approaches including erythropoietin, N-methyl d-aspartate antagonists, and gamma-aminobutyric acid, have shown some potential in preclinical acute stroke trials, the effectiveness of such treatments has not been demonstrated in clinical trials [21,22]. Thus, to improve functional recovery, the development of novel treatment strategies with an expanded therapeutic window after acute stroke is required. This review aims to provide a conceptual framework for considering the therapeutic targets and directions for treatment of brain dysfunction, with a particular focus on ischemic stroke.

## 2. Microglia Related Neuroinflammation in Ischemic Stroke

Microglia are the primary resident immune system cells, and they play a key role in the maintenance of the central nervous system (CNS) [6]. Microglia maintain the quiescent state, stimulating the typically thin ramified processes that occur in a normal healthy environment [23]. They recognize various external stimuli such as glycoproteins, aggregated proteins, cytokines, chemokines and the inflammatory microenvironment [24]. When microglia detect a broad range of stimuli from an infection, toxic chemical, ischemia or brain injury, they migrate into lesions and transform into several different functional activated forms with different morphologies, producing different cytokines and receptors [6,25]. Several studies have shown that microglia cells regulate the neuroinflammatory response after ischemic stroke [26,27]. After ischemic stroke, microglia cells are activated and release inflammatory cytokines such as interleukin-1β, tumor necrosis factor-α, interleukin-6 [28]. Microglia can adopt three different activation morphologies: M1 (classically activated macrophages), M2 (wound-healing macrophages) and regulatory macrophages via induction by different stimuli [29]. M1 activation of microglia occurs by induction of interferon-γ (IFN-γ) and tumor necrosis factor (TNF) and induces the secretion of pro-inflammatory mediators such as IL-6, IL-12 and IL-23 from microglia [30,31].

M2 activation of microglia is triggered by the cyclic AMP response element binding protein (CREB) and the C/EBPβ transcription factor, which not only induces the secretion of IL-4, IL-10, TGF-β and neuroprotective factors, but also stimulates the initiation of chronic inflammatory processes (tissue repair) that inhibit pro-inflammatory responses [32,33]

**Figure 1:**
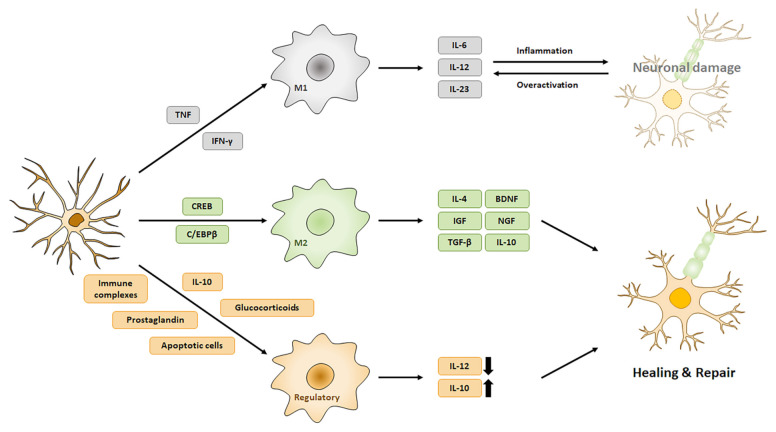
Microglia activation patterns and related factors. Neuroinflammation produce various environmental stimuli after ischemic stroke. Microglia change their morphology through different environmental stimuli and produce various pro- or anti-inflammatory factors.

Regulatory macrophages are involved in the late stage of the adaptive immune response. These cells arise via the stimulation of glucocorticoids (from adrenal cells in the hypothalamic–pituitary–adrenal (HPA) axis), immune complexes, prostaglandin, apoptotic cells and IL-10. After stimulation, these cells upregulate the production of IL-10 and downregulate IL-12, while also inhibiting the production of pro-inflammatory cytokines (Figure 1) [34]. Microglia activation has been correlated with the development of age-related neuroinflammatory neurodegenerative conditions including neuroinflammation Alzheimer’s disease, Parkinson’s disease, dementia correlated with the human immunodeficiency virus, and age-related macular degeneration (AMD) [23]. In aged human brains, age-related abnormalities in the cytoplasmic structure of sporadic microglial cells have been shown to appear. Such abnormalities are termed “microglial dystrophic” features and are characterized by reduced dendritic complexity with increased residual processes being observed, including tortuosity, cytoplasmic beading, and fragmentation, reflective of the ongoing cytorrhexis [35]. Microglia activation displays functional abnormalities, including increased levels of pro-inflammatory cytokines and reactive oxygen species, in addition to increased mitochondrial contents compared to young microglia [36].

Furthermore, it has been found that aged microglia express increased levels of inflammatory cytokines, such as interleukin-1β, tumor necrosis factor-α, and interleukin-6 [37,38]. These findings indicate that aged microglia may contribute to the neuroinflammation state, which is related to enhanced susceptibility of the aged CNS systemic environment to neurodegenerative disease.

The neurotoxic effects of microglia activation become progressively worse in neurodegenerative diseases. Inflammatory triggers mediate these neurotoxic effects both directly on the neurons and indirectly via the over-activation of microglia. Pro-inflammatory molecules produced from microglia enhance neurotoxicity in neurons, which results in the cumulative influence of environmental insults such as microglial activators (laminin, MMP3, etc.). These activators lead to severe neurotoxicity by over-activation of microglia in prolonged and lasting insults such as stroke [39]. Inflammation induces neurotoxicity in neurodegenerative diseases such as stroke and promotes microglial activation, leading to microglial dysregulation and over-activation. Microglia activation has a bi-lateral effect for neuronal survival and death in cerebral ischemia. In the degenerative environment of cerebral ischemia, several factors are recognized as pathogenic signals by microglia and these lasting pathogenic signals lead to progressive and cumulative neurotoxicity by microglial over-activation. Both the pro- and anti-inflammatory effects of microglia are necessary for initiating recovery systems following a stroke. However, the balanced regulation of microglial activation is poorly understood. Delineating the controlling mechanism of microglial activation may be necessary for improving therapy for stroke patients in future studies.

## 3. Pathophysiology and Biomarkers in Ischemic Stroke

The pathological mechanisms of stroke include energy failure, oxidative stress, disruption of the blood–brain barrier (BBB), neuro-excitotoxicity, brain inflammation, microglial activation, and endothelial injury. Previous clinical studies have emphasized the importance of understanding these mechanisms in order to diagnose and manage ischemic stroke [40,41,42].

Ischemic stroke is commonly characterized as the rapid interruption of blood supply, leading to failed neurological function resulting from oxygen deprivation and glucose delivery to the brain tissue [14,43]. During brain ischemia, cerebral blood flow is significantly decreased (by approximately 6–14 ± 2 mL/100 g/min) which leads to the induction of pathological processes [44]. During these processes, the ischemic penumbra exhibits structurally reversible features resulting from the ischemic event—generally defined as the hypoperfused area [45]-and represents a clinical target for ischemic stroke therapy. Stroke-induced angiogenesis occurs in the peri-infarct regions and ventricular/subventricular zone (V/SVZ) of the lateral ventricles [46]. Furthermore, stroke-induced neurogenesis occurs in the V/SVZ and in the subgranular zone of the dentate gyrus, a neural stem cell region [47]. In the V/SVZ region, neural stem cell proliferation generated by a stroke is involved in the stimulation of cerebral endothelial cells [46,48].

Biomarkers could be a valuable component of diagnostic assessment approaches and guide treatment and prognosis. Thus, stroke biomarkers could improve treatment by enabling the early diagnosis and facilitating sequential monitoring of patients, in addition to providing a rapid assessment of the severity of brain damage [49,50,51]. The use of these diagnostic biomarkers in the pathophysiological processes of a stroke would be particularly important in patients with non-localized or transient neurological symptoms [49,50,51]. An example of a glial-specific biomarker of acute brain injury is the S100 calcium-binding protein B (S100B), a common astrocytic marker [52] with important roles in Ca${}^{2+}$ homeostasis, astrocyte glutamate uptake, and neurite outgrowth stimulation [53]. However, S100B as a prospective marker is elevated in ischemic stroke, as is glutamate excitotoxicity and astrogliosis, which commonly occur in cases of trauma and traumatic brain injury [54,55]. Glial fibrillary acidic protein (GFAP) is a brain specific type III intermediate filament protein that is expressed by mature astrocytes and some other glial cells [56]. Several aspects of brain injuries and neurological disorders involve gliosis and the induction of GFAP, which has been proposed as a biomarker of glial injury in stroke [57,58]. As important indicators of brain damage, ubiquitin C-terminal hydrolase-L1, myelin basic protein, neuron-specific enolase have been studied [59,60,61,62]. In addition, fibronectin, D-dimer and von Willebrand factor have been associated with ischemic stroke and are known as biomarkers that are involved in coagulation and thrombosis [63,64,65]. Laskowiz et al. described a panel of diagnostic biomarkers in stroke including S100B, von Willebrand factor, matrix metalloprotease-9, B-type nerve growth factor and MCP-1 [66]. These are intimately involved in ischemic stroke and, therefore, can be used as potential biomarkers (Table 1).

During ischemic stroke, oxidative stress from free radicals, such as superoxide anions, hydroxyl radicals, hydrogen peroxide and nitric oxide, is closely related to brain tissue injury [67,68]. These free radicals, as potential indirect biomarkers of oxidative stress, are associated with increased Ca${}^{2+}$ levels, disruptions in mitochondrial integrity in ischemia and reperfusion, and—ultimately—lead to cell death [69,70,71]. Importantly, 8-hydroxy-2’-deoxyguanine, a product of DNA oxidation, has been widely proposed as a biomarker of oxidative damage in stroke patients [72,73]. Ischemic stroke is extremely complex and is reflected in post-ischemic inflammation, involving endothelial cells, astrocytes, microglia and neurons [74,75]. During ischemic inflammation, upregulation of Ca${}^{2+}$ levels, free radicals and secretion of proinflammatory cytokines such as interleukin-1, tumor necrosis factor-α and interleukin-1β, as well as neuroprotective factors including erythropoietin, transforming growth factor β1, and metallothionein-2, has been reported [76,77,78]. Biomarker measurement could be performed during the onset of initial symptoms, enabling expedited treatment of high-risk patients, potentially facilitating early stroke recovery. Presently, various ischemic stroke candidate biomarkers are being examined; however, none are currently being investigated in clinical trials and, thus, further studies are needed.

## 4. Application of Stem Cell Therapy in Stroke

Cell-based therapy is an emerging and promising new therapeutic strategy for enhancing tissue repair and neurological recovery in ischemic stroke patients [79,80]. The primary objective of cell-based therapies is to restore or replace damaged tissue with functional cells, with the final aim of integrating these cells with the remaining functional original cells and supporting the restoration of lost organ function. Recent studies have suggested that stem cells can increase the functional recovery of damaged tissue in post-ischemic stroke through various mechanisms, including stem cell secretion of neurotrophic factors, immunomodulation, stimulation of endogenous neurogenesis, and neovascularization [81,82,83,84,85,86,87].

Previous preclinical studies have reported that stem cell therapy may enhance functional recovery and behavioral improvements in animal models of stroke [82,88]. Numerous researchers have reported on the use of stem cells harvested from various sources including umbilical cord blood cells [89], adipose derived stem cells [90], amniotic fluid stem cells [91], bone marrow endothelial progenitor cells [92], multipotent progenitor cells [93], neural stem cells (NSCs) [94,95] and induced pluripotent stem cells [96]. Other investigators have selected mesenchymal stem cells (MSCs) for treatment of neurological disease because these cells are easy to collect in large numbers for clinical use. These stem cells have been transplanted through various routes in animal models of stroke, leading to recovery of brain impairment and dysfunctions. The therapeutic effects of MSC-based therapy for the treatment of stroke include the promotion of CNS plasticity and neurovascular remodeling, angiogenesis, and immunomodulation [91,97].

The interaction between transplanted MSCs and brain parenchymal cells promotes neurological recovery; this interaction is mediated by intercellular communication through extracellular vesicles released from stem cells [98,99]. Exosomes are an especially important class of extracellular vesicles that are released by MSCs. Previous studies have reported that MSCs release large numbers of exosomes, which leads to the integration of MSCs with other cells [100]. Exosomes are endosome-derived small membrane vesicles (30 nm to 100 nm in size) that are secreted into the extracellular space by cells [101]. They are located in the blood and cerebrospinal fluid, and play important roles in intercellular communication by transferring cargo between source and target cells [101,102]. Emerging data indicate that exosomes released from MSCs have therapeutic effects in stroke and traumatic brain injury by regulating the microenvironment surrounding the brain [103,104]. In addition, exosomes transfer their microRNAs (miRNAs) from mesenchymal stromal cells to neuronal cells [105], with MSC-derived exosomes including more than 700 miRNAs that interact with argonaute 2, a component of the RNA induced silencing complex [106,107]. The effects of manipulating MSC-derived exosomes to produce and transport increased levels of miRNAs in brain tissue replacement after stroke have been investigated both and [108]. Moreover, investigations into the advantages of exosomal therapies in promoting brain repair processes after ischemic stroke may enhance stroke recovery and lead to the development of novel therapies. Despite fascinating preclinical studies of the STAIR (stroke Therapy Academic Industry Roundtable) criteria using these cell types to repair brain damage, clinical trial is underway and is still the initial stages. Furthermore, stem cell-based therapies are associated with ethical issues, and transplantation of iPSC (induced Pluripotent Stem Cell) is accompanied with the risk of teratoma formation [109,110,111,112]. Thus, the precise mechanisms of the host’s response after treatment with these therapies, in addition to the fate of donor cells, require further investigation.

## 5. Imaging Techniques for Stem Cell Therapy

Current diagnostic tests for stroke rely on neurological assessments using various neuroimaging modalities. As stroke types (i.e., ischemic versus thrombotic) clearly differ, knowledge of these clinical differences is very important in stroke treatment approaches. Distinguishing between the clinical differences in stroke types has been facilitated by the application of diagnostic imaging systems including computed tomography (CT) and magnetic resonance imaging (MRI) [113]. Advances in imaging techniques to classify stroke have been rapid and, in part, prompted by the need for improved clinical approaches. Currently, imaging methods and other investigative methods are combined to facilitate the tracking of stem cell transplantation in stroke patients, including MRI, nuclear medicine imaging and OI (Optical Imaging) [114].

To adequately investigate stem cell therapies for neurological diseases in preclinical studies, it is very important to develop imaging technologies that are able to evaluate disease progression by “chasing” (i.e., tracking or monitoring) transplanted stem cells in recipients. The pathology of stroke and regeneration by transplanted stem cells develop through either endogenous or exogenous mechanisms, which can be assessed using various imaging technologies, including traditional tissue staining, immunohistochemistry, autoradiography, electrophysiological analysis, and molecular biological methods [115,116].

Most traditional evaluation methods, such as immunohistochemistry and molecular biological techniques, have been useful tools for assessing disease progress only in preclinical studies because these techniques are only able to analyze post-mortem or collected tissues. Many studies have described changes in infarct volume due to stroke in middle cerebral artery occlusion animal models using tissue staining with 2,3,5-triphenyltetrazolium chloride, and post-stroke neural cell loss evaluated using the “TUNEL” assay [90,93,117]. Some researchers have used electron microscopy as tool to observe the repair of the BBB (Blood-brain-barrier) system and to track transplanted stem cells [92,118,119]. However, these technologies only use post-mortem samples, not live animals or human subjects. Using molecular analyses, researchers can determine alterations in gene or protein responses induced by stroke, such as upregulation of GLT-1 and vascular endothelial growth factor, and reductions in peri-ischemic extracellular glutamate [120,121], in addition to reductions in the inflammatory responses [93,96] of stem cells. Immunofluorescence staining facilitates the visualization of the induction of several neuron types and different blood vessels [117,122,123].

In more recent stroke studies, the importance of developing more sensitive and non-invasive OI technologies has been highlighted. MRI and CT are the common imaging modalities used by clinicians. Fluorescence, bioluminescence and nanoparticle imaging have been developed to generate more sensitive data, support the diagnosis of ischemic stroke pathology, such as infarct size and hemorrhage, and the restore cerebral blood flow, and enable the tracking of engrafted stem cells using CT and MRI as non-invasive methods [124,125]. Accordingly, some researchers have used MRI [89,119,126] or positron emission tomography–CT [127,128] to evaluate infarct volume in live animals. Nanoparticle technology is able to detect and chase transplanted stem cells using MRI in live animals [119,129,130].

Superparamagnetic iron oxide nanoparticles (SPION) have been labeled with stem cells from various sources such as NSCs (Neural Stem cell), BMSC (Bone Marrow Stem Cell), MSCs (Mesenchymal Stromal Cell), embryonic stem cells (ESCs) in order to chase the survival, migration and integration of transplanted stem cells in animal ischemic stroke models via MRI [131,132,133,134,135,136,137,138]. Organic fluorescent nanoparticles with aggregation-induced emission also represent good tracking particles. MSCs have been transplanted into a rat ischemia model after fluorescent nanoparticles labeling. These nanoparticles have low cytotoxic effects and do not affect the normal function of MSCs [139,140]. These nanoparticles have been used to perform multiple tasks, not only related to detecting stem cells themselves, but also to delivering effective drugs. Recent studies have demonstrated that stem cells promote neurogenic, neuroprotective and vasculogenic properties, while they are also capable of producing therapeutic effects and stimulating the creation of a microenvironment, using nanoparticles, via the delivery the therapeutic agent, retinoic acid (RA) [141], antioxidant [142] and chemoattractant [143].

Fluorescence imaging visualizes light emitted by green fluorescent proteins or emission fluorescent dyes such as DiI, DiO and CMFDA (5-Chloromethylfluorescein diacetate) in anesthetized animals [144,145,146]. Bioluminescence imaging visualizes photons emitted by luciferase enzyme reactions in live animals. To enable bioluminescence imaging, however, stem cells must be genetically modified to express the luciferase enzyme before transplantation [147,148] Stem cells can be labeled with nanoparticles such as superparamagnetic iron oxide nanoparticles, quantum dots, and pebbles in order to chase transplanted stem cells using MRI [149,150,151]. One study suggested the use of NSCs for the treatment of neurological diseases in a nonhuman primate stroke animal model. In this study, magnetic labeled transplanted human NSCs, chased using MRI, survived for 2 years in the nonhuman primate brain without immunosuppression and, moreover, differentiated into neurons without tumorigenesis [94] (Table 1).

## 6. Outlook and Conclusions

Rapid advances are occurring in the development of imaging technologies, with the utilization of more sensitive invasive and non-invasive methods. It is necessary to develop multimodal imaging techniques for the diagnosis of stroke and to evaluate the effects of stem cell therapy. Combining multimodal imaging approaches has compensated for some of the limitations in the currently available methods. Although OI techniques provide direct evidence for the survival, migration, integration and differentiation of stem cells, obstacles to stroke therapy remain, including the development of higher-resolution probes and materials to label stem cells. Both the development and application of non-invasive OI techniques are necessary in order to fully understand stroke progression and the therapeutic potential of stem cells in clinical studies involving human subjects.

During a stroke event, microglia are activated and secrete cytokines, which lead to cell death. To prevent aggravation of the stroke, hyperactivation of microglia needs to be regulated. Thus, many researchers have examined the alterations of various genes in the ischemic region. Such genes are used as biomarkers for stroke diagnosis, which can prevent the serious deterioration of stroke through early diagnosis. Furthermore, biomarkers can represent the basis of stem cell-based therapy as a novel strategy for promoting neurological recovery.

## Figures and Tables

**Table 1:** Investigation of reported ischemic stroke Biomarkers.

Biomarker	Role in Ischemic Stroke	Description	Application to Ischemic Stroke	Reference
S100β	Glial damage	Calcium binding protein, regulation of cell cycle progression and differentiation.	Diagnosis, stroke severity	[152,153,154]
GFAP	Glia protein	Intermediate filament proteins of mature astrocytes	Diagnosis	[155,156]
MBP	Glial damage	The most abundant protein components of myelin in the CNS	Diagnosis	[157,158]
NSE	Neuronal damage	Neurotrophic and neuroprotective properties on a broad spectrum of CNS	Diagnosis	[159,160]
Fibronectin	Hemostasis	A glycoprotein of extracellular matrix, binds to integrins collagen, fibrin	Diagnosis, Stroke risk	[161,162]
D-dimer	Hemostasis	Fibrin degradation product	Diagnosis	[160,163]
vWF	Hemostasis	A blood glycoprotein involved in hemostasis	Diagnosis	[164,165]
MMP9	Inflammation	Zinc-metalloproteinases family involved in the degradation of the extracellular matrix	Diagnosis	[166]
MCP1	Inflammation	A small chemokine, recruits monocytes, memory T cells, and dendritic cells	Diagnosis	[167]
IL-6	Inflammation	A pro-inflammatory cytokine	Diagnosis	[168,169]
UCH-L1	deubiquitinating	Deubiquitinating enzyme, highly specific to neurons	Diagnosis	[155]

## Data Availability

Not applicable.

## Abbreviations

The following abbreviations are used in this manuscript:

S100β	S100 calcium-binding protein B
GFAP	Glial fibrillary acidic protein
MBP	Myelin Basic Protein
NSE	Neuron specific enolase
vWF	von Willebrand factor
MMP9	Matrix metallopeptidase 9
MCP-1	monocyte chemoattractant protein-1
IL-6	Interleukin 6
UCH-L1	Ubiquitin C-terminal hydrolase L1
